# Novel Flowable Hemostatic Agent ActiClot: Efficacy and Safety Assessment in Rat and Porcine Models

**DOI:** 10.3390/jcm13164770

**Published:** 2024-08-14

**Authors:** Hee-Jung Kim, Su-Kyoung Lee, Yun-Jeh Ko, Soo-Hyeon Jeon, Eun-Jin Kim, Oh-Hyeong Kwon, Yang-Hyun Cho

**Affiliations:** 1 Department of Thoracic and Cardiovascular Surgery, Korea University Anam Hospital, Seoul 02841, Republic of Korea; heejung440@hanmail.net; 2 Korea Artificial Organ Center, Korea University, Seoul 02841, Republic of Korea; lsk9236@naver.com; 3 Department of Polymer Science and Engineering, Kumoh National Institute of Technology, Gumi 39177, Gyeongbuk, Republic of Korea; yunjehko@gmail.com; 4 Theracion Biomedical Co., Ltd., Seongnam 13201, Gyeonggi, Republic of Korea; shjeon@theracion.com (S.-H.J.); ejkim@theracion.com (E.-J.K.); 5 Department of Thoracic and Cardiovascular Surgery, Samsung Medical Center, Sungkyunkwan University School of Medicine, Seoul 16419, Republic of Korea

**Keywords:** surgical bleeding control, hemostatic performance, carboxymethyl starch, safety assessment

## Abstract

**Background/Objectives**: This study evaluated the hemostatic performance and safety of ActiClot (ATC), a new flowable hemostatic agent, through in vivo tests. **Methods**: ATC was compared with the commercially available FLOSEAL^®^. ATC consists of carboxymethyl starch, thrombin, and sorbitol powders in Syringe I, and a calcium chloride solution in Syringe II. In vivo evaluation used rat liver bleeding and porcine heart bleeding models. Safety was assessed using a rat subcutaneous implantation model. **Results**: ATC significantly reduced hemostasis time (70.00 ± 7.35 s) compared to gauze control (240.63 ± 32.31 s) in the rat liver model, showing a 70% reduction. There was no significant difference between ATC and FLOSEAL^®^ (58.75 ± 13.42 s). In the porcine heart model, both agents achieved 100% hemostasis within 3 min, with no significant difference in success rates within 2 min (ATC 87.5%, FLOSEAL^®^ 75%). The gauze control group failed in all tests. The rat subcutaneous implantation model showed no visual ATC observation after 48 h, indicating biocompatibility, with no inflammation observed. **Conclusions**: ATC demonstrated effective hemostatic performance similar to FLOSEAL^®^ in two in vivo models, with faster hemostasis in the rat liver model. It also showed excellent safety and biocompatibility, indicating its potential for surgical and emergency bleeding control.

## 1. Introduction

Bleeding control is an indispensable aspect of patient care during surgical interventions and emergency procedures. The consequences of ineffective hemostasis can be severe, including increased transfusion requirements, prolonged operation times, escalated surgical costs, infections, and even potential mortality [1,2,3,4]. As a result, achieving successful hemostasis becomes a primary responsibility for medical personnel, leading to the exploration of various strategies and hemostatic agents. One such approach that has shown promise involves combining gelatin and thrombin in a flowable matrix, demonstrating active mechanisms in managing cases of active bleeding [1,5,6,7]. The slower the biodegradation of a hemostatic agent, the more likely it is to remain in the body and cause an inflammatory response [8,9]. The slower the biodegradation of a hemostatic agent, the more likely it is to remain in the body and cause an inflammatory response. The gelatin in FLOSEAL^®^ remains in the body even after 6 weeks and can cause side effects such as inflammation [10]. When a raw material is slowly biodegraded in the body, the residue can trigger a foreign body reaction, increasing the possibility of inflammation. Compared to gelatin products, polysaccharide powders are favored due to their fast degradation and minimal inflammatory response [11,12].

To overcome this limitation, we developed a novel approach that utilizes a plant-originated source, carboxymethyl starch, in conjunction with thrombin to create a flowable hemostatic agent. This innovative agent may offer the advantage of adaptability to both typical and deep bleeding sites while maintaining excellent safety profiles. Thus, the present study aims to comprehensively investigate the efficacy and safety of starch and thrombin-based flowable hemostatic agents.

## 2. Materials and Methods

### 2.1. Hemostatic Agent

**Figure 1:**
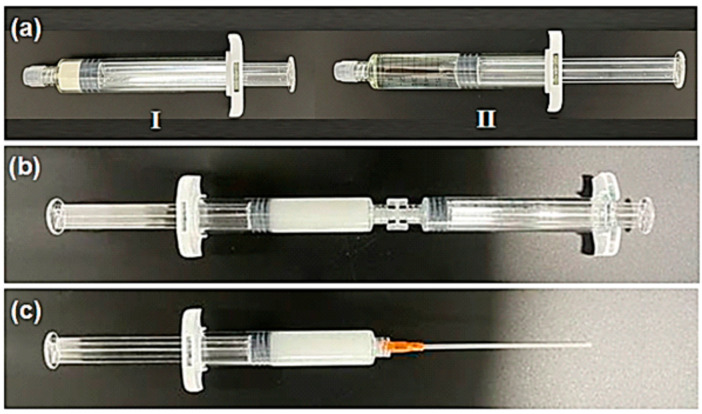
The ActiClot consists of two syringes (Syringe I and II, 15 mL) (**a**) and a connection hub. Syringe I contains carboxymethyl starch, thrombin, and sorbitol powders. Syringe II contains a calcium chloride solution. The ActiClot can be used simply by connecting and mixing both syringes using a hub (**b**) and a nozzle (**c**) without the process of reconstitution of thrombin or refilling it into another syringe.

In this study, the hemostatic performance and safety of ActiClot (ATC, Theracion biomedical, Seongnam, Republic of Korea) were confirmed by in vivo tests. The hemostatic performance of FLOSEAL^®^ was compared with in vivo tests. ATC is a flowable, paste-like matrix, morphologically similar to FLOSEAL^®^ (Baxter, Deerfield, IL, USA), SurgiFlo (Johnson and Johnson, New Brunswick, NJ, USA), and Collastat^®^ (Dalim Tissen Co., Ltd., Seoul, Republic of Korea), but there are differences in the materials constituting the hemostatic agent [1]. ATC consists of two syringes (Syringe I and II) and a connection hub. Syringe I contains carboxymethyl starch, thrombin, and sorbitol powders. Syringe II contains a calcium chloride solution. ATC can be used simply by connecting and mixing a syringe using a hub without the process of reconstitution of thrombin or refilling it into another syringe (Figure 1).

### 2.2. In Vivo Evaluation of Hemostasis

(1) Rat liver bleeding wound model test

Sprague Dawley (SD) rats (weight: 230~270 g, Daehan Biolink, Seoul, Republic of Korea) were used to compare the hemostatic performance of ATC and FLOSEAL^®^ (Baxter, USA). The animal model experiment was performed under the Institutional Animal Care and Use Committee of Konkuk University (No. KU21203). After the animal was anesthetized with an anesthesia machine (Compact 70, Aika, Tokyo, Japan) (isoflurane, Aesica Queenborough Ltd., Queenborough, UK), the fur was removed and an incision was made in the center of the abdomen. A 6 mm-deep wound was made on the left hepatic lobe of the liver with a 6 mm punch biopsy and bleeding was checked. The evaluation was performed in 8 parallel groups (8 rats per parallel group), and after recording the average hemostasis time, the sample was washed and a picture of the wound was taken. The control group consisted of rats treated only with gauze. All three rats were euthanized following the procedure.

(2) Porcine heart bleeding model test

The hemostatic performance of ATC and FLOSEAL^®^ was evaluated using pigs (Yorkshire Swine, XP-bio, Donghae, Republic of Korea) and performed at the Laboratory Animal Center, Korea University College of Medicine (KOREA-2022-0150). The included pigs were all female and weighed more than 50 kg. The animals were pre-anesthetized (Zoletile 50, 0.1 mL/kg) and connected to a ventilator, and respiratory anesthesia (isoflurane, 1 L/min, Aesica Queenborough Ltd., UK) was performed to maintain anesthesia. Blood pressure and heart rate were observed within the normal range during the experiment. Anticoagulants or antiplatelet agents were not used, and antibiotics (Cefazolin) and muscle relaxants (Vecaron or Esmeron) were administered to prevent infection and spontaneous breathing of the animals. After sterilizing the animal’s chest with povidone–iodine, a thoracotomy was performed along the midline. For the hemostatic test, three separate incisions were sequentially performed on the myocardium. Each incision was 1 cm in length and did not perforate the heart chamber. The incisions were made parallel to the coronary artery to avoid arterial bleeding. After each incision, a hemostatic agent was applied, and the bleeding condition was checked at 1, 2, and 3 min. The order of hemostatic agents was chosen randomly (Gauze, ACT, FLOSEAL). The bleeding condition was visually assessed, and hemostasis success was evaluated using a three-grade bleeding scale: (1) complete hemostasis; (2) oozing—slow, minor bleeding that does not accumulate rapidly; and (3) active bleeding—continuous, rapid bleeding requiring immediate intervention [11,13]. Therefore, we obtained two comparative hemostatic tests from one pig. After surgery, each pig was euthanized.

### 2.3. In Vivo Evaluation of Safety by Rat Subcutaneous Implantation Model

The SD rats (Sprague Dawley rats, Daehan Biolink, Republic of Korea) weighing 230 to 270 g were used to evaluate the safety of ATC. The skin was incised after the animal was anesthetized with an anesthesia machine (Compact 70, isoflurane, Aika, Japan). The ATC (0.2 mL) was inserted on the right side of the incision and after the administration of antibiotics (cefazoline) and pain reliever (carprofen). 

#### Techniques for Inflammation Evaluation

(1) Histological evaluation: Surrounding tissues, including the implant, were collected at specified intervals (2, 4, 10, and 14 days) post implantation.
(2) Tissue fixation: The collected tissue was fixed by immersing it in a 10% formalin solution.
(3) Tissue processing: The fixed tissue was embedded in paraffin and processed following standard protocols.
(4) Tissue sectioning: The paraffin-embedded tissue was sectioned into 5 µm-thick slices using a microtome.
(5) Staining and observation: Hematoxylin and eosin (H&E) staining and Masson’s trichrome staining were performed on the tissue sections.

Three animals were evaluated for each time point.

Inflammation was assessed by observing the stained tissue under a microscope: determining the amount of residual material in the sample tissue and assessing the grade by counting the number of infiltrated inflammatory cells. The histological evaluation score and microscopic scoring for article absorption are described in Supplementary Tables S1 and S2 [14,15,16].

### 2.4. Statistical Analysis

In vivo hemostatic performance in rat model: Data are presented as means ± standard deviation. Statistical analyses were performed using KyPlot version 2.0 (KyensLab, Inc., Tokyo, Japan). Parametric Student’s *t*-tests and one-way analysis of variance (ANOVA) with Tukey’s post-hoc test were used to determine statistical significance, with a threshold set at *p* < 0.05.

Porcine heart bleeding model test: Categorical variables are presented as frequencies and percentages. Chi-square or Fisher’s exact tests were used to evaluate the efficacy of complete hemostasis at 2 min following the hemostasis procedure. Statistical significance was determined with a threshold of *p* < 0.05.

## 3. Results

### 3.1. In Vivo Hemostatic Performance in Rat Model

**Figure 2:**
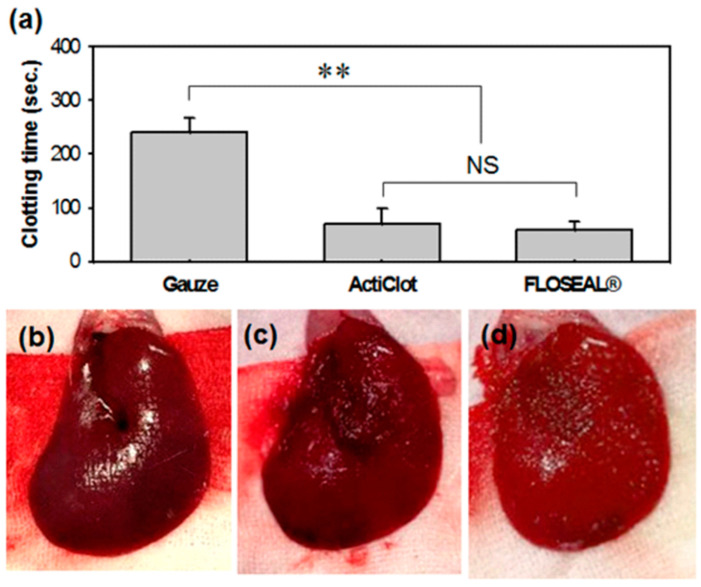
In vivo hemostatic efficacy on rat liver injury model. (**a**) Hemostasis time measurement of the gauze, ActiClot, and FLOSEAL^®^ groups and additional bleeding check results after hemostatic agent removal; (**b**) gauze, (**c**) ActiClot, and (**d**) FLOSEAL^®^ groups. (n = 8, ** *p* < 0.05, NS means not significant)**.**

The hemostasis performance using a rat liver injury model was compared by measuring the time to hemostasis of the gauze (control group), ATC, and FLOSEAL^®^ groups. The hemostasis time of the ATC was 70.00 ± 7.35 s on average, which was shorter than that of the gauze (240.63 ± 32.31 s). By using ATC, the hemostasis time was shortened by about 70%. There was no statistically significant difference from the FLOSEAL^®^ (58.75 ± 13.42 s) group (Figure 2a). In addition, when the ATC and the FLOSEAL^®^ were removed after hemostasis, there was no additional bleeding (Figure 2b–d).

### 3.2. In Vivo Hemostatic Performance in Porcine Model

**Figure 3:**
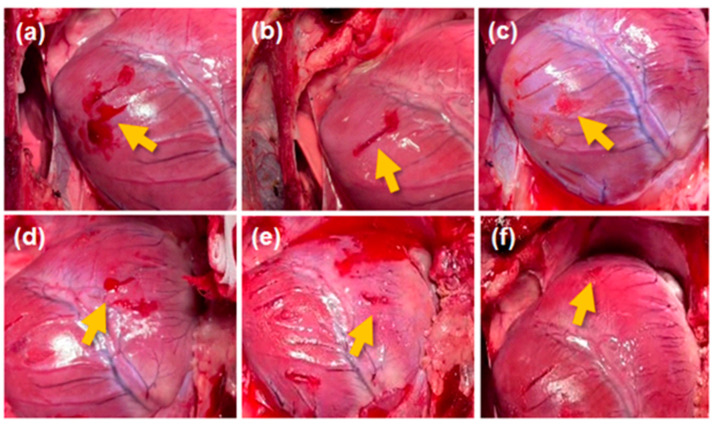
In vivo hemostasis confirmation after 2 min of compression in the porcine heart muscle incision model. The left (**a**–**c**) and right (**d**–**f**) ventricles of the porcine heart treated with (**a**,**d**) cotton gauze, (**b**,**e**) ActiClot, and (**c**,**f**) FLOSEAL^®^. The yellow arrow indicates the hemostatic condition after applying hemostatic methods.

Four pigs were used for the test (mean weight: 53.2 ± 2.6 kg). The hemostasis performance regarding the porcine heart muscle incision model of the gauze (control group), ATC, and FLOSEAL^®^ groups was evaluated by evaluating hemostasis time and bleeding pattern (Figure 3). The ATC and FLOSEAL^®^ groups succeeded with hemostasis (100%) within 3 min of compression, and the success rate of hemostasis within 2 min was also 87.5% for the ATC and 75% for the FLOSEAL^®^ groups, and there was no statistical significance between the two groups (*p* = 1.0). However, the gauze group failed to stop bleeding in all tests compared to hemostatic agents (Table 1, control versus ATC, control versus FLOSEAL^®^).

### 3.3. In Vivo Evaluation of Safety by Rat Subcutaneous Implantation Model

**Figure 4:**
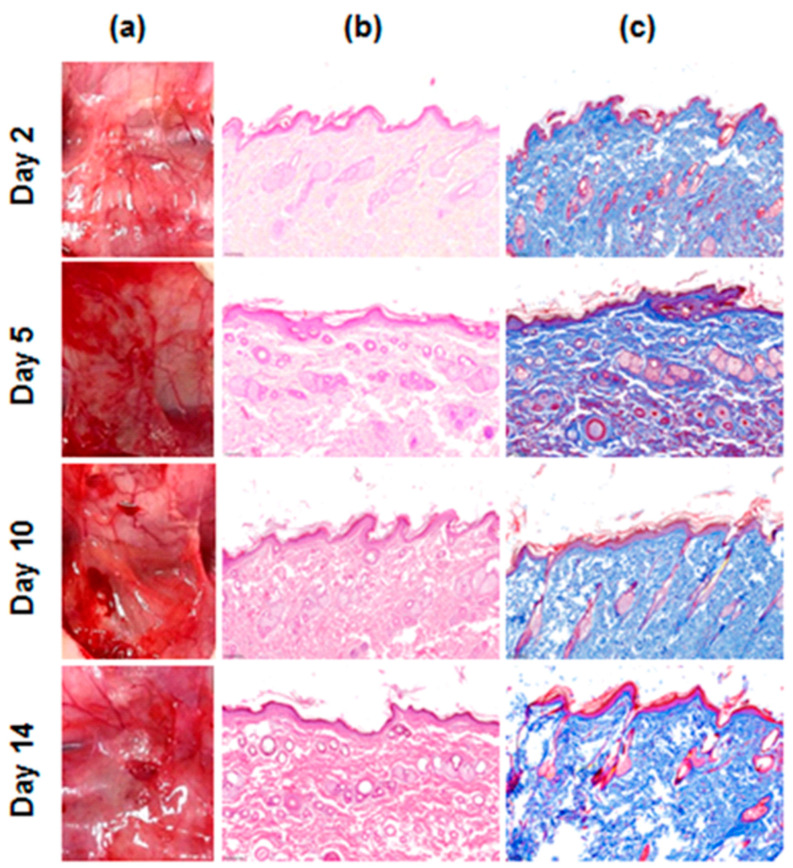
In vivo safety evaluation of the ActiClot matrix in the rat model. (**a**) Photographs of the site after subcutaneous implantation and (**b**) the photomicrographs H&E, ((**b**), pink; cytoplasm, dark blue; nuclei), Masson’s trichrome ((**c**), dark red; keratin and muscle fibers, pink; cytoplasm, light blue; collagen, dark blue; nuclei) staining of the ActiClot implanted tissue.

The biological safety of hemostatic agents is essential for medical uses. The evaluation was investigated in the rat model over time after subcutaneous transplantation with visual observation and histology (Figure 4). Remains of the ATC agent were not observed after 48 h of transplantation (Figure 4a). Inflammation did not occur at the transplant site at any point during the transplantation period (Figure 4b,c).

## 4. Discussion

The representative materials used as hemostatic agents are gelatin, collagen, oxidized cellulose, calcium alginate, chitosan with sponge, fabric, film, granules, powders, and paste types [1,5,6,7,8,9,11,17,18,19,20,21]. Gelatin is an animal-derived protein-based hemostatic agent used to stop bleeding and promote blood clotting. However, it has limitations due to ethical concerns regarding animal sourcing and potential allergic reactions. Collagen promotes blood clotting. It is highly compatible with tissues and forms a strong bond with human tissues [1,5,6,7,8,9]. Oxidized cellulose has high absorbency and helps to stop bleeding and promote blood clotting [21]. It is recognized as a renewable natural material that may minimize the risk of infection. Calcium alginate is a hemostatic agent derived from seaweed containing calcium components [19]. It has excellent absorbency and is effective in stopping bleeding and aiding wound healing. Chitosan is a natural polymer extracted from crustaceans such as shrimp and crabs [19,20,21]. It is used as a hemostatic agent to stop bleeding and promote wound healing. It also possesses antimicrobial effects, reducing the risk of infection. Various types of polymers are used as hemostatic agents. These materials are applied to wounds or incisions to stop bleeding and have high absorbency and mechanical strength.

Among them, one of the dominant hemostatic biomaterials, gelatin, is derived from animals, raising ethical concerns about its production (Gelfoam^®^, Surgifoam^®^, and Surgiflo™). Some individuals may experience allergic reactions to gelatin, leading to potential side effects. The use of gelatin hemostatic agents may carry a risk of infection. In some cases, gelatin hemostatic agents may not completely stop bleeding, necessitating the use of other hemostatic techniques. Efforts through research and the development of alternative materials may address these issues and lead to improvements in hemostatic agents. Polysaccharide-based hemostatic agents, composed of cellulose and starch, offer several advantages over gelatin-based agents. Being derived from natural plant sources, these substances typically have a lower risk of infection and immunologic reactions compared to animal-based products [22,23]. Additionally, their higher degradation speed is associated with a reduced risk of mass effects on adjacent organs [10,11]. This makes them a safer alternative in various surgical settings. In this study, we investigated the in vivo hemostatic performance and safety of a novel bioabsorbable, plant-based hemostatic agent, ATC, in rat and porcine models. Hemostasis is a critical process to control bleeding during surgical procedures, and the development of effective hemostatic agents can significantly improve patient outcomes.

In the rat liver injury model, ATC demonstrated a remarkable hemostatic performance compared to conventional gauze. The average hemostasis time of ATC was significantly shorter (70.00 ± 7.35 s) than that of gauze (240.63 ± 32.31 s). This indicates that ATC can substantially reduce the time required to achieve hemostasis, which is beneficial for minimizing blood loss during surgeries. The ability of ATC to shorten the hemostasis time by approximately 70% compared to gauze highlights its potential as an efficient hemostatic agent. Notably, there was no statistically significant difference in hemostasis time between the ATC and the commercially available FLOSEAL^®^ group (58.75 ± 13.42 s). This suggests that ATC is as effective as FLOSEAL^®^, a widely used hemostatic agent, in achieving hemostasis in the rat liver injury model. The comparable performance of ATC to FLOSEAL^®^ indicates that our novel hemostatic agent can be a promising alternative to existing commercially available options. Moreover, the absence of additional bleeding after removing ATC and FLOSEAL^®^ further supports their efficacy in achieving stable and lasting hemostasis. This characteristic is crucial during surgical procedures, where any post-hemostasis bleeding can lead to complications and may necessitate additional interventions.

To further assess the hemostatic performance of ATC, we conducted experiments in a porcine model using heart muscle incisions. In this model, both ATC and FLOSEAL^®^ demonstrated a high success rate (100%) in achieving hemostasis within 3 min of compression. Additionally, ATC showed a comparable success rate (87.5%) to FLOSEAL^®^ (75%) in achieving hemostasis within 2 min. These results indicate that ATC performs equally as well as FLOSEAL^®^ in achieving rapid hemostasis in a porcine model, demonstrating its potential for application in larger animal models and eventually in clinical settings. In contrast, the gauze group failed to achieve hemostasis in all tests compared to the hemostatic agents. This outcome highlights the limitations of conventional gauze in controlling bleeding effectively, especially in situations where a rapid and reliable hemostatic agent is required.

We evaluated the safety of ATC using a rat subcutaneous implantation model (Figure 4). Throughout the transplantation period, we did not observe any visual remains of ATC at the transplant site after 48 h, indicating that the agent was resorbed or degraded, likely reducing the risk of long-term adverse effects. Importantly, no signs of inflammation were observed at the transplant site (Figure 4b,c), suggesting that ATC did not elicit an inflammatory response, which is crucial for the successful integration of a hemostatic agent in clinical practice.

There are several limitations of the study. (1) The use of animal models, although common in preclinical research, may not fully represent human physiology. (2) The relatively small sample size in the porcine model might have implications on the statistical power and generalizability of the results. (3) The study primarily focused on short-term outcomes, such as immediate hemostasis and early safety evaluations. The absence of long-term data restricts our ability to assess potential delayed reactions or chronic effects of ATC on tissue healing and biocompatibility. (4) The partial support from the ATC manufacturer introduces the potential for bias in study design, data analysis, and interpretation. While we maintained research integrity, external funding sources should be acknowledged to ensure transparency. Due to the limitations of the animal study mentioned above, further research with human subjects is essential to evaluate the hemostatic efficacy in various surgical fields and the inflammatory response.

## 5. Conclusions

Our study demonstrates that ATC exhibited excellent in vivo hemostatic performance in both rat and porcine models. It significantly shortened the time required for hemostasis compared to conventional gauze and performed on par with the commercially available hemostatic agent, FLOSEAL^®^. Moreover, ATC showed a high success rate in achieving hemostasis in porcine heart muscle incisions. The absence of inflammation and visual remains at the transplant site in the rat subcutaneous implantation model supports the biological safety of ATC. Taken together, these findings suggest that ATC has the potential to be a promising hemostatic agent for use in various surgical procedures. Further investigations and clinical trials are warranted to confirm its efficacy and safety in human applications.

## Figures and Tables

**Table 1:** Hemostatic status at 2 min after application of hemostatic agents on porcine heart muscle incision model (n = 4, LV: left ventricles, RV: right ventricles, A: active bleeding, B: oozing, C: complete hemostasis).

Groups	LV				RV			
	#1	#2	#3	#4	#1	#2	#3	#4
Gauze	A	A	A	A	A	A	A	A
ActiClot	C	C	B	C	C	C	C	C
FLOSEAL^®^	C	C	C	C	B	C	B	C

At 3 min after applying ActiClot or FLOSEAL^®^, hemostasis was observed in all incision sites. But the control group failed to achieve hemostasis.

## Data Availability

The data that support the findings of this study are available on request from the corresponding author.

## Supplementary Materials

The following supporting information can be downloaded at: https://www.mdpi.com/article/10.3390/jcm13164770/s1, Table S1: Histological evaluation system; Table S2: Microscopic Scoring Criteria for Article Absorption.
