# Supplementary material for: Novel Flowable Hemostatic Agent ActiClot: Efficacy and Safety Assessment in Rat and Porcine Models
Source: J Clin Med. 2024 Aug 14;13(16):4770. doi: 10.3390/jcm13164770 (PMC11355466; doi:10.3390/jcm13164770)
Supplement: Supplementary file 1 [file jcm-13-04770-s001.zip › jcm-3096928-supplementary.pdf]

Supplementary Table S1. Histological evaluation system

Cell type/ response	Score				
	0	1	2	3	4
Polymorphonuclear cells	0	1-5/hpf *	6-10/hpf	Heavy infiltrate	Packed
Lymphocytes	0	1-5/hpf	6-10/hpf		
Plasma cells	0	1-5/hpf	6-10/hpf		
Macrophages	0	1-5/hpf	6-10/hpf		
Giant Cells	0	1-2/hpf	3-5/hpf		Sheets
Necrosis	0	Minimal	Mild	Moderate	Severe

*hpf=high-powered field or equivalent area per microscopic assessment (40x objective)

Supplementary Table S2. Microscopic Scoring Criteria for Article Absorption

Score	Description
0	Not absorbed: Approximately more than 90% of the material/void is present.
1	Partially absorbed: Approximately 11-89% of the material/void is present.
2	Essentially absorbed: Approximately 10% or less of the material/void is present.
3	Completely absorbed: No extracellular material/void is presend and evidence of tissue reaction resolution is observed.
NP	Implant not present: No extracellular material/void is present and no evidence of tissue reaction resolution is observed (this site will be subtracted from total number of siter exmined.)
